# Effect of CBM1 and linker region on enzymatic properties of a novel thermostable dimeric GH10 xylanase (Xyn10A) from filamentous fungus *Aspergillus fumigatus* Z5

**DOI:** 10.1186/s13568-018-0576-5

**Published:** 2018-03-21

**Authors:** Youzhi Miao, Yanqiong Kong, Pan Li, Guangqi Li, Dongyang Liu, Qirong Shen, Ruifu Zhang

**Affiliations:** 1 Jiangsu Provincial Key Lab for Organic Solid Waste Utilization, National Engineering Research Center for Organic-based Fertilizers, Jiangsu Collaborative Innovation Center for Solid Organic Waste Resource Utilization, Nanjing Agricultural University, Nanjing, 210095 China; 2 Key Laboratory of Microbial Resources Collection and Preservation, Ministry of Agriculture, Institute of Agricultural Resources and Regional Planning, Chinese Academy of Agricultural Sciences, Beijing, 100081 People’s Republic of China; 3 College of Resources & Environmental Science, Nanjing Agricultural University, Nanjing, 210095 China

**Keywords:** Xylanases, Thermostability, Biofuel, Lignocellulose, CBM1 and linker

## Abstract

Xylanase with a high thermostability will satisfy the needs of raising the temperature of hydrolysis to improve the rheology of the broth in industry of biomass conversion. In this study, a xylanase gene (*xyn10A*), predicted to encode a hydrolase domain of GH10, a linker region and a CBM1 domain, was cloned from a superior lignocellulose degrading strain *Aspergillus fumigatus* Z5 and successfully expressed in *Pichia pastoris* X33. Xyn10A has a specific xylanase activity of 34.4 U mg^−1^, and is optimally active at 90 °C and pH 6.0. Xyn10A shows quite stable at pHs ranging from 3.0 to 11.0, and keeps over 40% of xylanase activity after incubation at 70 °C for 1 h. Removal of CBM1 domain has a slight negative effect on its thermostability, but the further cleavage of linker region significantly decreased its stability at high temperature. The transfer of CBM1 and linker region to another GH10 xylanase can help to increase the thermostability. In addition, hydrolase domains between the two Xyn10A proteins naturally formed a dimer structure, which became more thermostable after removing the CBM1 or/and linker region. This thermostable Xyn10A is a suitable candidate for the highly efficient fungal enzyme cocktails for biomass conversion.

**Electronic supplementary material:**

The online version of this article (10.1186/s13568-018-0576-5) contains supplementary material, which is available to authorized users.

## Introduction

Xylanases have been used in many industrial processes as the additives to improve the quality of baked goods and animal feeds as well as to bleach kraft pulp. In biomass conversion, xylanases also play a crucial role to synergistically deconstruct lignocellulose with cellulases and release soluble sugars from polysaccharide of xylan (Inoue et al. 2015). Besides the pre-treatment of biomass and the design of efficient enzyme cocktails, the whole industrial process also need high thermostable enzymes, which have many benefits of increased specific activity, stability, prevention of growth of contaminants, and increased mass transfer rate due to lower fluid viscosity at high substrate concentrations at high temperature, to save the cost (Kubicek and Kubicek 2016). To this end, the searching of high thermostable xylanases is valuable.

Xylanases have the molecular function in hydrolyzing xylan, which is the second most abundant polysaccharide after cellulose (Pauly and Keegstra 2008), and mainly constituted by a linear backbone of β-d-xylopyranosyl residues linked by β-1,4-glycosidic bonds. The complete degradation of xylan actually needs various enzymes, including the essential endo-β-1,4-xylanases and β-xylosidases, also including accessory enzymes, such as acetyl xylan esterases, α-l-arabinofuranosidases, α-glucuronidases and phenolic acid esterases, due to the substitution of xylosyl residues in the xylan backbone by acetyl, arabinosyl, glucuronysyl and 4-*O*-methylglucuronycyl residues (Ebringerova and Heinze 2000). In CAZY database (http://www.cazy.org), xylanases are categorized into different families (GH5, GH8, GH10, GH11, GH30 and GH43) based on their structural similarities (Lombard et al. 2014), where the GH10 and GH11 are found to be widely distributed in fungi. Meanwhile, the carbohydrate-binding modules (like families CBM1, CBM2, CBM6 and CBM22) are usually located in the protein structure of GH10 xylanases linked by a flexible linker region. These xylanases-related CBMs were found to have different polysaccharides-binding functions, such as CBM1, binding crystalline cellulose, CBM6 and CBM22, binding insoluble xylan. To data, analysis of the effect of CBMs and linker region on the thermostability of GH10 xylanases has yielded some inconclusive results (Liu et al. 2015; Matsuzawa et al. 2016; Meng et al. 2015), however, the mechanisms by which non-catalytic domains contribute to the thermostability has not yet been determined.

Filamentous fungi are used for biomass conversion in industry for their excellent properties in secreting all required enzymes (cellulases, hemicellulases and ligninases). Therefore, a screening for thermostable xylanases in filamentous fungi will directly meet the demands when compared to bacteria-derived enzymes, which sill exit unsolved difficulty of expressing them in filamentous fungi. *Aspergillus fumigatus* Z5 is a superior natural biomass degrader with genome sequence available (Liu et al. 2011, 2013; Miao et al. 2015a). This strain can be cultured at high temperature of more than 50 °C and has been used in straw composting usually. In this study, we describe the cloning, expression and functional characterization of the thermostable Xyn10A of *A. fumigatus* Z5, which belongs to GH10 family and has a CBM1 domain linked on C-terminus by a Ser/Thr-rich linker. The effect of CBM1 and linker region on the properties of Xyn10A and Xyn10B are also investigated and discussed.

## Materials and methods

### Strains and growth conditions

*Aspergillus fumigatus* Z5 (CGMCC Accession No. 3309, China General Microbiology Culture Collection Center, Genome GenBank accession AZZA01000000) is stored in our lab and used for the study of lignocellulose degradation. The cultivation was performed according to Miao et al. (2015a). Briefly, 1 × 10^7^ fresh conidia was added into 200 ml of Mandels’ salt solution supplemented with 2% (w/v) oat spelts xylan (Sigma, USA), and then incubated for 20 h at 50 °C and 150 rpm. The mycelia were then harvested and washed thoroughly with sterile water to be stored at − 80 °C for RNA extraction.

*Pichia pastoris* X33 (Invitrogen, USA) was used as the gene expression host. Plasmid construction and storage was based on the *Escherichia coli* Top10 (stored in our lab) cultivated in LLB (Low-salt Luria–Bertani) medium (1% peptone, 0.5% yeast extract, 0.5% NaCl, pH7.0). YPDS medium (2% peptone, 1% yeast extract, 2% glucose, 1 M sorbitol, pH 6.0) was used for transformants screening. BMGY/BMMY (2% peptone, 1% yeast extract, 1.34% YNB, 4 × 10^−5^% biotin, 1% glycerol or 0.5% methanol, pH 6.0) was used as the growth/induction medium for enzyme production.

### cDNA synthesis, plasmid construction and enzyme preparation

Xylan-induced mycelia of strain Z5 was grinded in liquid nitrogen for total RNA extraction using RNeasy Plant Mini Kit (Qiagen, Germany) combined with the RNase-Free DNase set (Qiagen, Germany) according to the manufacturer’s instructions. A 1 μg mass of total RNA with good quality was used as the template for cDNA synthesis using PrimeScript™ RT-PCR Kit (TAKARA, China).

The open reading frames (ORFs) of these xylanases encoding genes, excluding their native signal sequences, were amplified by PCR using the synthesized xylan-induced cDNA as the template. All primers are listed in Additional file 1: Table S1. PrimeSTAR HS DNA polymerase (TAKARA, China) was used for high-fidelity PCR amplifications. Gene fragments were inserted into pPICZαA/B using T4 DNA Ligase (TAKARA, China) and verified by DNA sequencing, the correct plasmids (pPICZαB-Xyn10A, pPICZαB-Xyn10AdC, pPICZαB-Xyn10AdLC, pPICZαA-Xyn10B and pPICZαB-Xyn10BaLC) were linearized with PmeI (New England Biolabs, China) and then transformed into *P. pastoris* X33 by electroporation (Gene Pulser Xcell™ Electroporation System #165-2660, Bio-Rad, USA) according to the manufacturer’s protocol. Transformants were screened in the YPDS plates containing 100 μg ml^−1^ of zeocin. The correct transformants (*P. pastoris* X33-Xyn10A, *P. pastoris* X33-Xyn10AdC, *P. pastoris* X33-Xyn10AdLC, *P. pastoris* X33-Xyn10B, *P. pastoris* X33-Xyn10BaLC) were verified by PCR, and then cultured in 100 ml BMGY medium in a 500 ml flask for 20 h at 30 °C and 200 rpm. The cultures were centrifuged for 5 min at 3000 rpm, and the supernatants were discarded, then *P. pastoris* cells were transferred into the fresh BMMY medium for enzyme inductions. Every 24 h, 100% methanol was added to a final concentration of 1% in the medium. After 96 h, the supernatants were collected by centrifugation and proteins were extracted by the 80% of ammonium sulfate. The transformants used for further study were finally confirmed by the extracellular enzyme activities and SDS-PAGE analysis. Enzyme purifications were carried out according to the procedure described previously (Miao et al. 2015b).

### Enzyme activity assay

Xylanase activity was measured by DNS method (Linton and Greenaway 2004) with some modifications. 5 μg of each purified xylanase was incubated with 1 ml of substrate solution, consisting of 1% (w/v) oat spelts xylan in sodium acetate buffer (50 mM, pH 6.0), at each optimal reaction temperature for 10 min, then reaction was terminated by adding 1 ml of 3,5-dinitrosalicylic acid (DNS) and boiled for 10 min. The concentration of produced reducing sugars was determined by measuring the absorbance at 520 nm. d-Xylose (Sigma, China) was used as the control. One unit of enzyme activity was defined as the amount of enzyme required to release 1 μmol of reducing sugars from the substrate in 1 min.

### Characterization of the expressed xylanases

To determine the optimal reaction temperature of these expressed xylanases to the substrate of oat spelts xylan, the purified xylanase activities were detected as described above but at different temperatures ranging from 20 to 100 °C. Their thermostabilities were determined by incubating these enzymes at temperatures ranging from 20 to 100 °C for 6 h, then the residual enzyme activities were measured in each sampling time (10, 30 min, 1, 2, 4, 6 h).

The optimal pH for each enzyme was determined by incubating each xylanase with the substrate dissolved in an appropriate buffer at different pH values: 50 mM citrate buffer (pH 2.0–6.0), 50 mM PBS buffer (phosphate-buffered saline, pH 6.0–8.0), and 50 mM glycine–NaOH buffer (pH 8.0–11.0), at the optimal temperature for 10 min. Enzyme activity was determined as described above. For pH stability, the purified xylanases were pre-incubated in the different pH buffers for 1 h at 4 °C followed by activity determination at the optimal conditions.

Determination of the kinetic parameters for the purified xylanases was carried out under optimal conditions for 10 min using oat spelts xylan/xylan from beechwood at concentrations ranging from 2.5 to 20 mg ml^−1^. The reaction rate versus the substrate concentration was plotted, and the data were fitted to the Michaelis–Menten equation.

### Protein assay, SDS-PAGE and zymogram analysis

Protein extraction was carried out by ammonium sulfate precipitation. The clear supernatant was slowly added to 80% (w/v) ammonium sulfate on ice while stirred at low speed on a magnetic stirrer simultaneously. After incubating at 4 °C for 16 h, the sample was centrifuged at 10,000 rpm for 10 min at 4 °C, and the supernatant was decanted off. The protein pellet was resuspended in the sterile distilled water. Protein concentration was determined using a Micro BCA protein assay kit (Beyotime, China) according to the manufacturer’s instructions.

Sodium dodecyl sulfate–polyacrylamide gel electrophoresis (SDS-PAGE) was performed according to the description in Laemmli’s research (Laemmli 1970) on a 10% (w/v) polyacrylamide gel with a protein marker of PageRuler Prestained Protein Ladder (Fermentas, China) using a Mini-PROTEAN Tetra Cell Systems (Bio-Rad, USA). For zymogram analysis, protein samples were buffered by the loading buffer (16% (v/v) glycerol, 10% SDS, 250 mM Tris–HCl (pH 6.8) and 0.05 mg ml^−1^ Bromophenol Blue) without pre-heating, and then separated by the SDS-PAGE. After that, the gel was washed twice (30 min each) using phosphate-citrate buffer (pH 6.0) containing 25% isopropanol, followed by twice washing (30 min each) using the same buffer but without isopropanol. Then, the gel was covered on a xylan-containing agar plate (1% oat spelts xylan, 1.5% agar), and incubated at 50 °C for 30 min. the resulted agar plate was then stained by 0.5% congo red and destained by 1 M NaCl.

### Thin-layer chromatographic analysis

To investigate the final products from hydrolyzing oligosaccharides by Xyn10A, thin-layer chromatography (TLC) was used for sugar separation as described in our previous article (Miao et al. 2015b). Briefly, the hydrolyzed products were separated by a solvent system consisting of *n*-butanol–acetic acid–water (3:2:2, v/v), and subsequently visualized by spraying with a mixture of methanol and sulfuric acid (9:1, v/v) and heated at 60 °C for 20 min. Xylobiose, xylotriose and xylopentaose were used as the oligosaccharides for test.

## Results

### Characterization of the expressed Xyn10A

**Fig. 1:**
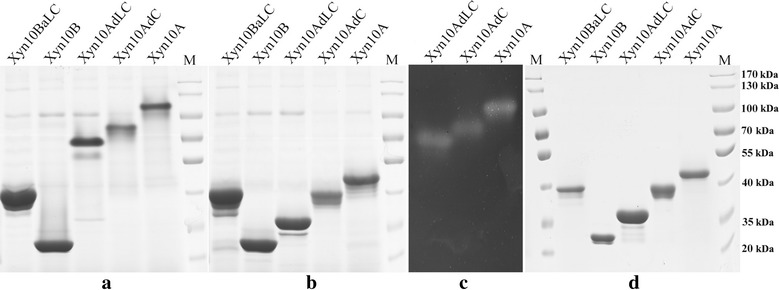
SDS-PAGE and zymogram analysis of the expressed xylanases. **a** These crude xylanases, Xyn10A, Xyn10AdC, Xyn10AdLC, Xyn10B and Xyn10BaLC (expressed in *P. pastoris* X33) were buffered and separated by SDS-PAGE but without pre-heating. **b** These crude xylanases, Xyn10A, Xyn10AdC, Xyn10AdLC, Xyn10B and Xyn10BaLC (expressed in *P. pastoris* X33) were buffered and separated by SDS-PAGE with heating treatment at 100 °C for 5 min before loading. **c** Zymogram analysis for Xyn10A, Xyn10AdC and Xyn10AdLC. **d** SDS-PAGE of the purified xylanases (Xyn10A, Xyn10AdC, Xyn10AdLC, Xyn10B and Xyn10BaLC). M, protein marker

Previous study indicated that *A. fumigatus* Z5 is a superior degrader for lignocellulosic biomass (Liu et al. 2011), and Xyn10A (Y699_04481) was identified as the most abundant secreted xylanase in the culture when strain Z5 was induced by xylan (Miao et al. 2015b). In this study, the whole gene fragment excluding an original signal peptide of 19 amino acids (AAs), was amplified from the xylan-induced cDNA of strain Z5 using the primers shown in Additional file 1: Table S1, and was then inserted into pPICZαB to obtain the expression vector of pPICZαB-Xyn10A, which was next transformed and successfully expressed in *P. pastoris* X33. SDS-PAGE (Fig. 1) shows that the expressed and purified Xyn10A has a molecular weight of approximately 50 kDa, which is relatively higher than the predicted 40.3 kDa. In the treatment where the protein sample of Xyn10A was buffered by 10% (m/v) SDS but without the preheating (100 °C for 5 min), SDS-PAGE showed that Xyn10A actually existed as a dimer in the gel (Fig. 1a, b). The dimer band was showed to be able to degrade xylan inside the xylan-containing plate to form a clear activity band in zymogram analysis (Fig. 1c), in contrast, the monomer produced by the preheating (70 °C for 5 min) in zymogram analysis could not be renatured after the SDS-PAGE. This result indicates that the dimer structure of Xyn10A is more beneficial for protein renaturation in zymogram analysis, and possibly has a function of keeping enzyme’s thermostability.

**Fig. 2:**
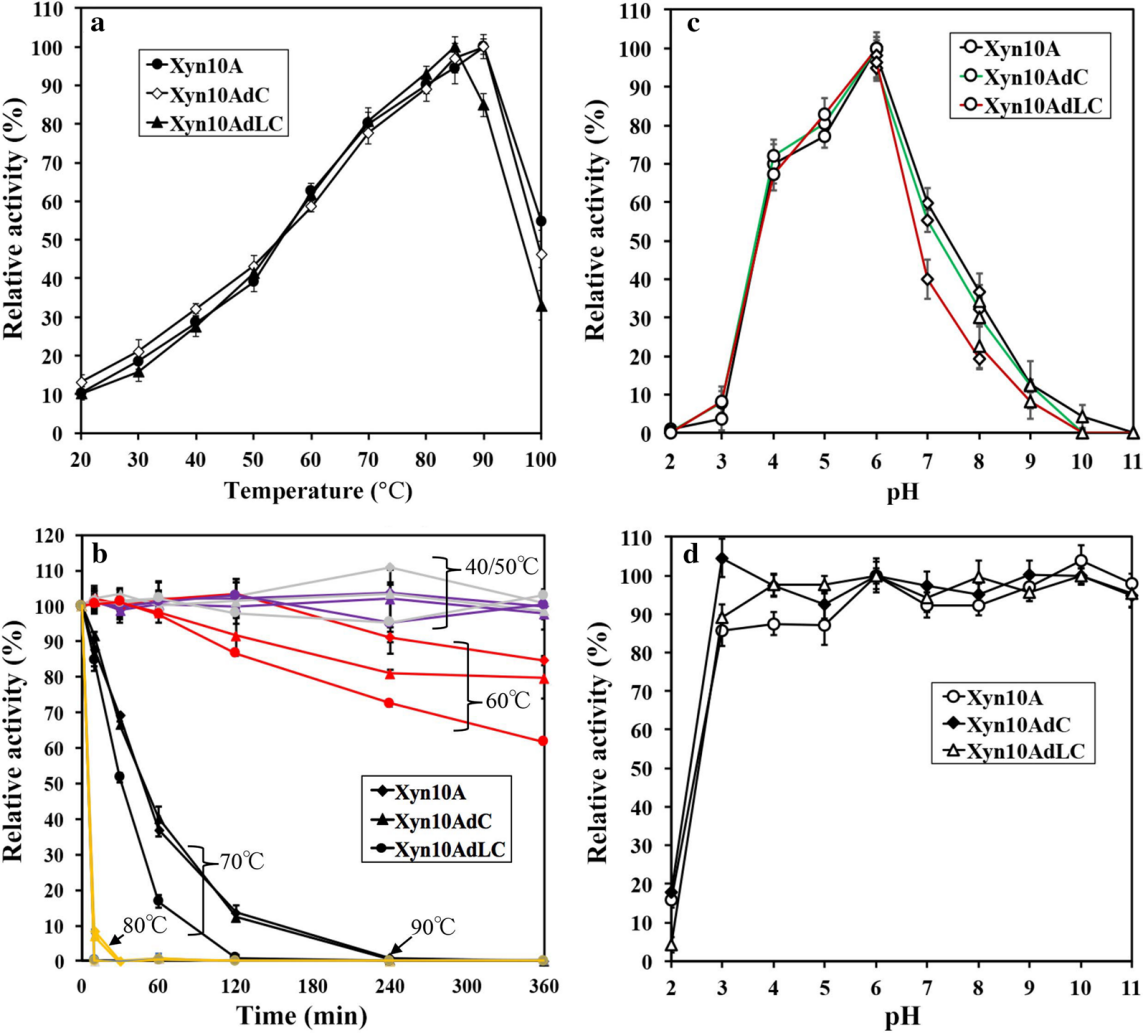
Temperature and pH properties for Xyn10A, Xyn10AdC and Xyn10AdLC. **a** Effects of the different temperatures on xylanase activities of the three purified xylanases (Xyn10A, Xyn10AdC and Xyn10AdLC). **b** Thermostability of the three purified xylanases (Xyn10A, Xyn10AdC and Xyn10AdLC), which were incubated in different temperatures until 6 h, and then xylanase activities were determined in six sampling points (0, 10, 30 min, 1, 2, 4, 6 h). Grey color represents their performances at 40 °C, purple color represents 50 °C, red color represents 60 °C, black color represents 70 °C, yellow color represents 80 °C, blue color represents 90 °C. **c** Effect of the pH on xylanase activities. pH buffers include 50 mM citrate buffer (circle, pH 2.0–6.0), 50 mM PBS buffer (diamond, pH 6.0–8.0), and 50 mM glycine–NaOH (triangle, pH 8.0–11.0); **d** pH stability of the three expressed xylanases (Xyn10A, Xyn10AdC and Xyn10AdLC). Each xylanase was incubated in the solutions with different pHs for 1 h at 4 °C, and then residual xylanase activity was determined. The bars stand for the standard errors of three replicates

The optimal reaction temperature for the substrate of oat spelts xylan was determined (Fig. 2a), and results showed that Xyn10A reached the highest xylanase activity at 90 °C. Even though at 100 °C, it still kept approximately 50% of xylanase activity in reaction time of 10 min. Thermostability experiment (Fig. 2b) showed that Xyn10A was quite stable in 6 h when incubation temperature not high than 60 °C. At 70 °C, this enzyme could retain over 40% of xylanase activity in 1 h. The maximal xylanase activity towards oat spelts xylan occurred at pH 6.0, and xylanase activity decreased rapidly by the pH increasing, indicating that Xyn10A is an acidic xylanase (Fig. 2c). pH stability (Fig. 2d) shows that Xyn10A was stable in various solutions with pH ranging from 3.0 to 11.0, however it lost nearly all xylanase activity at pH 2.0.

**Fig. 3:**
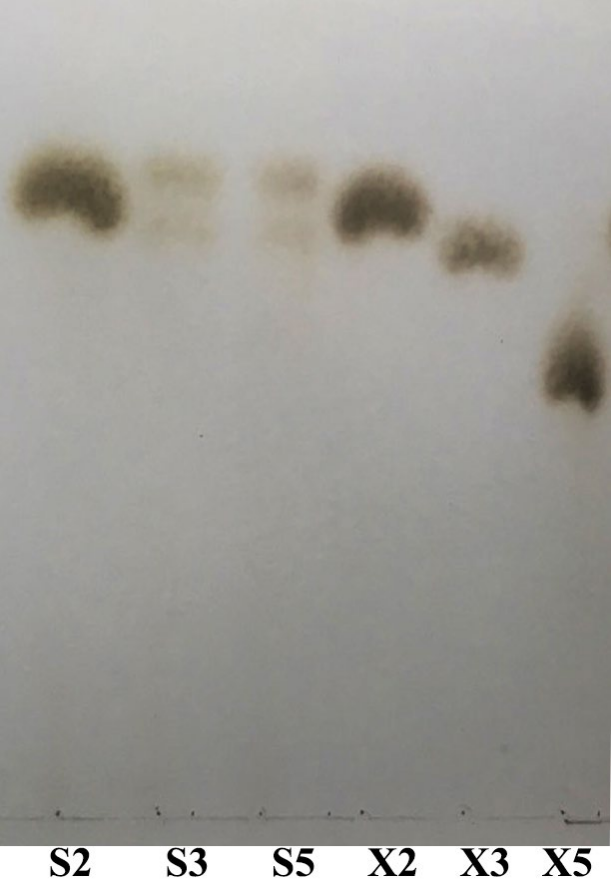
TLC analysis of the hydrolyzed products by Xyn10A. X2, the standard xylobiose; X3, the standard xylotriose; X5, the standard xylopentaose. S2, S3 and S5 are respectively the final products by Xyn10A after hydrolyzing xylobiose, xylotriose and xylopentaose at 50 °C for 10 min

In TLC analysis (Fig. 3), Xyn10A could hydrolyze xylopentaose and xylotriose into the final products of xylobiose and xylose. As same as other xylanases, xylobiose was not the direct substrate of this thermostable GH10 xylanase. The K_m_ value and specific activity of the purified Xyn10A towards oat spelts xylan were determined to be 1.56 mg ml^−1^ and 34.4 U mg^−1^, respectively.

### Effect of non-catalytic structures on the properties of Xyn10A

**Fig. 4:**
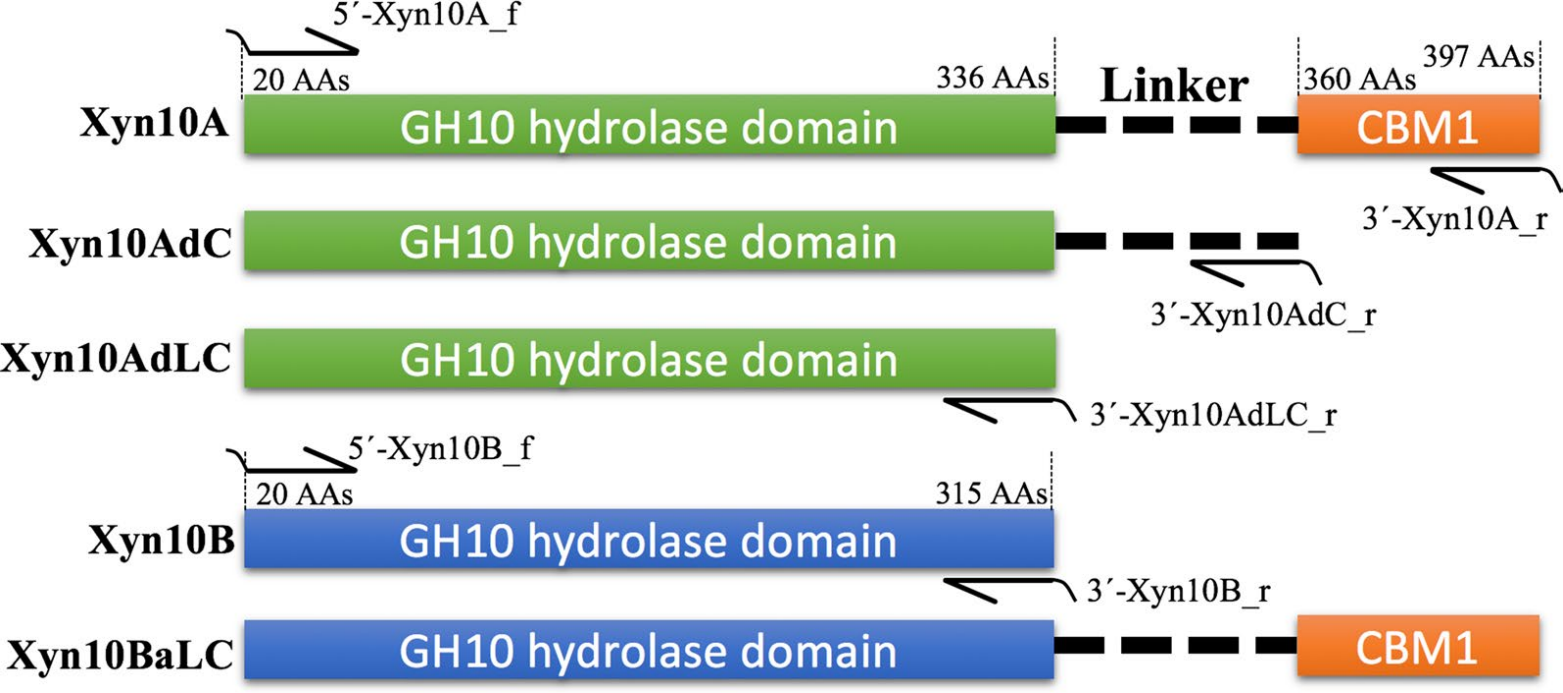
Enzyme structures of the five expressed xylanases in this study. Xyn10A has three different structures, including a GH10 hydrolase domain, a linker region and a CBM1. CBM1 was removed in Xyn10AdC, and in Xyn10AdLC, both CBM1 and linker region were removed. Xyn10B was engineered by adding of Xyn10A-derived CBM1 and linker on its C-terminus to obtain the Xyn10BaLC. The length of each domain was annotated by their original position of amino acids in *A. fumigatus* Z5. The expression primers were also indicated in the figure

Structure analysis based on the gene sequence of *Xyn10A* in *A. fumigatus* Z5 genome (GenBank Accession AZZA01000000) indicated that there are three different structures (Fig. 4) located in Xyn10A, including a hydrolase domain, a linker region and a CBM1 domain. CBM1 is responsible for cellulose binding function, and is linked to GH10 hydrolase domain by the linker region. To investigate the effect of these two non-catalytic structures on the thermostability of Xyn10A, the expression vectors (pPICZαB-Xyn10AdC and pPICZαB-Xyn10AdLC) were successfully constructed and expressed in *P. pastoris* X33. SDS-PAGE (Fig. 1) showed that Xyn10AdC (CBM1 removed) and Xyn10AdLC (CBM1 and linker removed) have the molecular weight of approximately 40 and 35 kDa respectively, which are also relatively higher than their predicted values. In zymogram analysis, these two engineered enzymes also formed dimers in the gel (Fig. 1a, b), and only the dimers could be renatured to show the clear activity bands (Fig. 1c), which indicates that the forces holding the dimer come from the interactions between two hydrolase domains.

Removal of CBM1 from Xyn10A (Xyn10AdC) did not change the optimal reaction temperature of 90 °C, but the further cleavage of linker region (Xyn10AdLC) caused a decreased optimal reaction temperature of 85 °C (Fig. 2a). Thermostability experiments (Fig. 2b) demonstrated that these three proteins (Xyn10A, Xyn10AdC and Xyn10AdLC) roughly had similar stability and could keep nearly all xylanase activities in 6 h below 60 °C. When incubated at the temperature of 90 °C, they would quickly lose activities in 10 min. The differences happened at temperatures of 60, 70 and 80 °C. For example, At 70 °C, Xyn10A and Xyn10AdC have similar thermostability, but Xyn10AdLC lost the activity more quickly. At 80 °C, Xyn10A and Xyn10AdC kept about 8% activities after 10 min incubation, but Xyn10AdLC lost 100% xylanase activity. At 60 °C, however, Xyn10A had an obviously higher thermostability than Xyna10AdC, and they were both more stable than Xyn10AdLC. These results indicate that CBM1 has only a slight effect on the thermostability, but further cleavage of linker region significantly decreases the thermostability of Xyn10A.

**Fig. 5:**
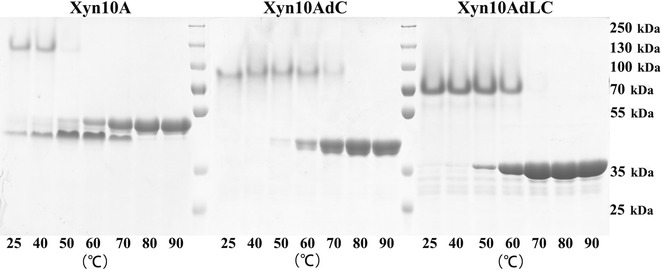
Effect of temperatures on the dimer structures of Xyn10A, Xyn10dC and Xyn10AdLC. Each purified enzyme was buffered using protein loading buffer, and then preheated at different temperatures (25, 40, 50, 60, 70, 80 and 90 °C) for 5 min respectively, subsequently separated by SDS-PAGE and visualized by coomassie brilliant blue staining. Protein marker used PageRuler™ Plus Prestained Protein Ladder (Fermentas)

As showed above, two hydrolase domains of each Xyn10A formed a dimer structure, meanwhile, the monomer of Xyn10A was also been detected to be existed as a little part at room temperature (Fig. 5). The dimer structure could be disrupted by high temperature treatments (Fig. 5), for example to Xyn10A, incubation at 50 °C for 5 min could largely make the dimer structure being separated and further no any detectable bond of dimer structure by SDS-PAGE if the temperature equal to/higher than 60 °C. However, for Xyn10AdC and Xyn10AdLC, the obvious protein bonds of dimer structures could still be detected by SDS-PAGE when treated at 70 °C for 5 min. This result showed that remove of CBM1 and linker actually increased the interactions between two hydrolase domains to keep the dimer structure more resistant to high temperature. In addition, dimer structures were not significantly affected by different salt concentrations, as showed in Fig. 1a, b [crude enzymes all having high concentration of (NH_4_)_2_SO_4_] and Fig. 5 (purified enzymes dissolved in 50 mM, pH 5.5 sodium acetate buffer). It should be reasonable that the flexible structure of CBM1 and linker located in Xyn10A had different conformations if without substrate for binding in culture, and this resulted in an interference to the interactions between two hydrolase domains as a form like space affect.

For optimal pH and pH stability, Xyn10A, Xyn10AdC and Xyn10AdLC all had the same optimal pH of 6.0, and were stable at pHs ranging from 3 to 11, but lost all xylanase activity at pH 2.0 (Fig. 2c, d). These results suggest that CBM1 and linker region do not affect the optimal pH and pH stability of Xyn10A.

### Thermostability transferring to Xyn10B by adding the CBM1 and linker of Xyn10A

**Fig. 6:**
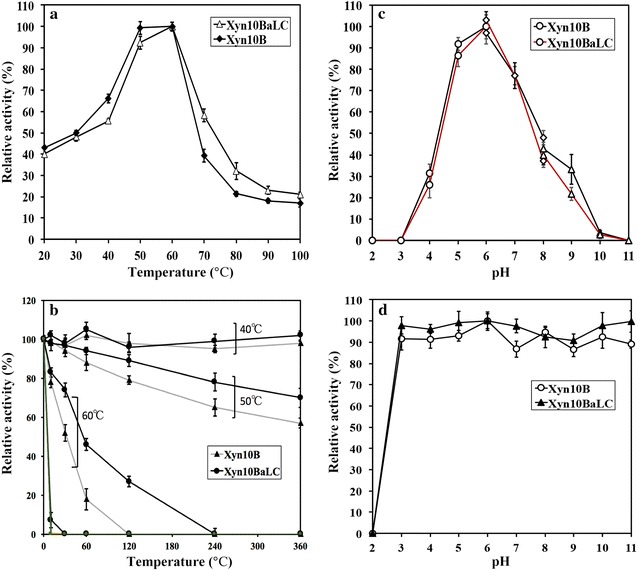
Temperature and pH properties for Xyn10B and Xyn10BaLC. **a** Effects of the different temperatures on xylanase activities of Xyn10B and Xyn10BaLC. **b** Thermostability of Xyn10B and Xyn10BaLC, which were incubated in different temperatures until 6 h, and then xylanase activities were determined in 6 sampling points (0, 10, 30 min, 1, 2, 4, 6 h). **c** Effect of the pH on xylanase activities. pH buffers include 50 mM citrate buffer (circle, pH 2.0–6.0), 50 mM PBS buffer (diamond, pH 6.0–8.0), and 50 mM glycine–NaOH (triangle, pH 8.0–11.0); **d** pH stability of Xyn10B and Xyn10BaLC. Each xylanase was incubated in the solutions with different pHs for 1 h, and then residual xylanase activity was determined. The bars stand for the standard errors of three replicates

Xyn10B (Y699_06333) was previously identified as another secreted GH10 family endo-β-1,4-xylanase by strain Z5, however, it only contains a hydrolase domain (Miao et al. 2015b). Here, the whole non-catalytic structure (CBM1 + linker region) of Xyn10A was engineered to be added to Xyn10B (producing Xyn10BaLC, Fig. 4) for investigating the contribution of CBM1 and linker to the thermostability of a different xylanase. Xyn10BaLC showed a larger molecular weight (> 40 kDa) than that of Xyn10B (< 35 kDa) in SDS-PAGE (Fig. 1). For the optimal reaction temperature, previous study (Miao et al. 2015b) reported that Xyn10B reached the highest xylanase activity towards the substrate of 1% oat spelts xylan at 60 °C, in this study, the addition of CBM1 and linker caused a small shift of the optimal reaction temperature to higher temperature, and increased the activities of Xyn10B at high temperatures (≥ 70 °C) (Fig. 6a). Thermostability experiment (Fig. 6b) showed that when temperature was higher than 70 °C, both Xyn10B and Xyn10BaLC lost activities in 10 min, but at 60 °C, Xyn10BaLC was relatively more stable than Xyn10B. These results indicated that the CBM1 and linker from Xyn10A really enhance the thermostability of another GH10 xylanase (Xyn10B).

Consistent with the Xyn10A, the added CBM1 and linker region in Xyn10BaLC did not affect the optimal pH (6.0) and pH stability (Fig. 6c, d). Both Xyn10B and Xyn10BaLC remained stable at pHs ranging from 3.0 to 11.0, and lost all xylanase activities at pH 2.0.

## Discussion

Enzymatic hydrolysis of hemicellulose is a complex process, in which multi-enzymes are required. Endo-β-1,4-xylanase plays the most important role for its function of breaking xylan backbone, and therefore be considered as a key enzyme for biomass conversion in industry. In microbes, fungi are excellent degraders of lignocellulosic biomass, such as *Neurospora crassa* (Phillips et al. 2011), a model organism for the study of biomass degradation mechanisms, and *Trichoderma reesei* (Martinez et al. 2008), being widely used in industry to produce enzymes for lignocellulosic biomass degradation. These well-studied filamentous fungi, however, can only grow normally below 30 °C, which may cause that their secreted xylanases usually have an optimal reaction temperature about 50 °C (Polizeli et al. 2005). Obviously, large amount of xylanases will be needed in biomass conversion, and the property of high thermostability could significantly save the cost for the longer working life and higher enzyme activity. Until now, thermostable xylanases have been mainly discovered in bacteria, such as *Thermotoga thermarum* (Shi et al. 2013), *Geobacillus thermoleovorans* (Sharma et al. 2007), *Acidothermus cellulolyticus*, *Enterobacter* sp. and *Clostridium* sp. (Bhalla et al. 2013), having the optimal temperatures ranged from 75 to 100 °C. However, the application of bacterial-derived enzymes as complements to fungal enzyme cocktails is severely limited by the still unsolved difficulty of expressing them in filamentous fungi. *A. fumigatus* Z5 was previously isolated from the compost heaps of crop straws and its genomic sequence was determined (Miao et al. 2015a). The genes encoding Xyn10A, Xyn10B and Xyn10C (three different xylanases) were identified in Z5’s genome. In the same species, such as *A. awamori*, *A. fischeri*, *A. kawachii*, *A. nidulans*, *A. oryzae*, *A. niger* and *A. terreus* xylanases were also expressed and purified, but only have the low optimal temperatures ranging from 40 to 60 °C (Polizeli et al. 2005). The protein of Xyn10A had several similar homologs (> 70%) when blasted against the NCBI database (http://www.ncbi.nlm.nih.gov), however, it was firstly reported in this study as a high thermostable GH10 xylanase with an optimal temperature of 90 °C in *Aspergillus* species. It will be suitable for adding this thermostable GH10 xylanase into enzyme cocktails by expressing it in an efficient protein expression system of *A. niger* (Wanka et al. 2016) or other industrial strains like *T. reesei*. If being considered, the potential of this enzyme still could be increased, such as enhancing the stability at high temperatures (> 80 °C), or improving the xylanase activity at low temperatures to make this enzyme work efficiently in a wide range of temperatures.

Xyn10A was predicted to have multiple protein domains, which included a 317 AAs hydrolase domain, a 24 AAs linker region and a 37 AAs CBM1 domain. CBM1 domain has a function of cellulose-binding (Boraston et al. 2004). With the help of CBM1, Xyn10A could get close to lignocellulosic biomass and bind the crystalline cellulose inside to degrade xylan around (Miao et al. 2017). From this aspect, CBM1 domain and linker region should play an important role during the effect on Xyn10A’s stability through the interactions of the basic amino acid chains or 3D structures, and the fact is that removal of CBM1 and linker from Xyn10A do decrease its ability to resist high temperature (> 70 °C). In natural degradation model, CBM1 domain binds the surface of crystalline cellulose by the thermodynamic forces (Nimlos et al. 2012), meanwhile the linker region is stretched to allow the function of the hydrolase domain. Badino et al. (2017) reported that different linker modifications could change the catalytic activity and cellulose affinity of cellobiohydrolase Cel7A from *Hypocrea jecorina* when hydrolyzing crystalline cellulose, which showed the possibility that the glycosylated linker region joined in affinity to substrate and further affect enzymes’ catalytic activities. However, in degradation of pure xylan, CBM1 do not have a specific-binding substrate (Miao et al. 2017), so the whole tension structure above would not exist. Protein simulation did not show a stable structure of Xyn10A that might support the possibility that CBM1 and linker would directly form a strong interaction with hydrolase domain to help its stabilizing if without substrate for binding. However, one reported crystal structure of GH10 xylanase from *Clostridium thermocellum*, containing a xylan-binding domain (XBD, CBM22 family), showing that The XBD had interactions with hydrolase domain, including 12 hydrogen bonds and 100 non-polar contacts (Najmudin et al. 2010). Also, some reports, that remove of XBD (CBM6-36 family) decreased thermostability of GH10 xylanase (Liu et al. 2015) and the adding could improve the enzyme’s thermostability (Matsuzawa et al. 2016), suggested the thermostabilizing function of XBD probably due to the interactions between XBD and hydrolase domain. Different to XBD, CBM1 domain cannot bind solid xylan, and may cause a relatively different conformation of Xyn10A with those XBD-containing xylanases when degrading the solid xylan. Although we cannot give that the interactions between CBM1/linker and hydrolase domain affected the thermostability of Xyn10A, it’s quite possible. Our previous result (Miao et al. 2017) showed that remove of CBM1 domain or together with linker region both significantly increased the xylanase activity of Xyn10A, indicating that the interference of CBM1 and linker to hydrolase domain actually existed. When the hydrolase domain is not protected by the linker region, it will be more prone for unfolding by high temperature. Compared to XBD-containing xylanases, in CBM1-containing xylanase of Xyn10A, the linker seems to perform a more important role than CBM1 domain in the probable interactions with hydrolase domain, which also been confirmed in a CBM13-containing xylanase of *Streptomyces* sp. S27 (Li et al. 2009). Thus, protein modification (glycosylation) was considered as a possible factor to affect the thermostability of Xyn10A for multi-glycosylation sites being predicted to be located on the linker region (data not shown). The observed molecular weights of Xyn10A, Xyn10AdC and Xyn10AdLC were all significantly higher than their predicted ones, indicating the modification of these enzymes when expressed in *P. pastoris* X33. However, Ribeiro et al. (Ribeiro et al. 2014) reported that the deglycosylation did not change the thermostability of a CBM1-containing GH10 xylanase from *Malbranchea pulchella*, which might reflect that glycosylation did not act as a main factor in the effect of linker on the thermostability of Xyn10A or divergences existed by species.

Xyn10A, Xyn10AdC and Xyn10AdLC all had the dimer structures in the solution at the normal temperature, which indicated that the dimer was actually formed between two hydrolase domains. For this reason, the two joined hydrolase domains probably have some interferences from each other when hydrolyzing the substrate of xylan. Together with the fact that the dimers of Xyn10A, Xyn10AdC and Xyn10AdLC all became largely separated to release each monomer when temperature above 50 °C, This may support some indirectly explanation why quite low percentages of xylanase activities were observed when temperatures below 50 °C compared to those values at high temperatures. Dimerization could rigidify this enzyme and caused a reduced enzyme activity. Another hand, the dimerization is likely to be a protection mechanism against the high temperature. As the temperature increasing, the catalytic activity of Xyn10A would be up-regulated because of the increasement of its thermal movement, when the temperature higher than 70 °C, the dimer began to separate and the protein structure would be unfolded step by step. In this process, the forces holding dimer of Xyn10A between the two hydrolase domains should be responsible for the stability of the whole protein structure at high temperatures, thus indirectly contributing to the catalytic activity of Xyn10A. It is certain that the dimer structure interferes the enzyme function of Xyn10A, however, it may keep this enzyme-structure more stable and its long-term existing in the environment. Filamentous fungi evolved to keep a balance between activity and stability of Xyn10A possibly for the more efficient degrading lignocellulose. Interestingly, when CBM1 and linker removed, the more stable dimer structure seemed not enough to recover the decrease of thermostability resulted by remove of the CBM1 and linker. Therefore, further study to the bio-function of this dimeric Xyn10A will be attractive.

In conclusion, a highly thermostable Xyn10A was cloned, expressed and characterized in this study. Natural Xyn10A formed a dimer structure between the two hydrolase domains. The contained CBM1 and linker region were confirmed to have a significant effect on the thermostability of Xyn10A. These non-catalytic structures (CBM1 and linker) were also demonstrated to be able to enhance other xylanases’ thermostability. Xyn10A is a suitable candidate to be added into the high efficient fungi enzyme mixtures for biomass conversion.

## Additional file

**Additional file 1: Table S1.** Primers used for enzyme expressions.

## Abbreviations

GH: glycoside hydrolase
CBM: carbohydrate-binding module
Xyn10A: a GH10 family xylanase from *A. fumigatu*s Z5
Xyn10AdC: CBM1 removed Xyn10A
Xyn10AdLC: CBM1 and linker region removed Xyn10A
Xyn10B: another GH10 family xylanase from *A. fumigatu*s Z5
Xyn10BaLC: CBM1 and linker region (from Xyn10A) added Xyn10B
