# Supplementary material for: Effect of CBM1 and linker region on enzymatic properties of a novel thermostable dimeric GH10 xylanase (Xyn10A) from filamentous fungus Aspergillus fumigatus Z5
Source: AMB Express. 2018 Mar 21;8:44. doi: 10.1186/s13568-018-0576-5 (PMC5862715; doi:10.1186/s13568-018-0576-5)
Supplement: Supplementary file 1 — Additional file 1: Table S1. Primers used for enzyme expressions. [file 13568_2018_576_MOESM1_ESM.docx]

**Table S1 Primers used for enzyme expressions**

| **Names** | **Sequences** |
| --- | --- |
| **Xyn10A series** |  |
| 5´-Xyn10A_f/5´-Xyn10AdC_f/5´-Xyn10AdLC_f | AAAACTGCAGGAGCTGGCCTGAACACAGCAG |
| 3´-Xyn10A_r/3´-Xyn10BaLC_r | CTAGTCTAGATCACAGGCACTGTGAGTACCAG |
| 3´-Xyn10AdC_r | CTAGTCTAGATCAAGTAGGGTCCGTACCTCCTG |
| 3´-Xyn10AdLC_r | CTAGTCTAGATCAAAGACCCGCCATCAGGCC |
| **Xyn10B series** |  |
| 5´-Xyn10BaLC_f | CCGGAATTCGGCGTGATCGACGAACGC |
| 3´-Xyn10BaLC_fusion_r**^a^** | CCGGAGCCGCTTGCTCCGAGAGCAGCAATGATGGCATTATACG |
| 5´-Xyn10BaLC_fusion_f | CGTATAATGCCATCATTGCTGCTCTCGGAGCAAGCGGCTCCGG |

^a^ _fusion, the primer for fusion PCR that adds CBM1 domain on the corresponding enzyme;

PstI site (CTGCAG), EcoRI site (GAATTC) and XbaI site (TCTAGA) were introduced into primers.
